# Managing silent threats: Unveiling gangrenous complication in acute emphysematous cholecystitis

**DOI:** 10.1016/j.radcr.2024.01.084

**Published:** 2024-02-17

**Authors:** Majid Asgari Mehr

**Affiliations:** Radiology Department, Holbæk Hospital, Smedelundsgade 60, 4300 Holbæk, Denmark

**Keywords:** Acute emphysematous cholecystitis, Gangrenous Cholecystitis, Computed tomography scan, Abdominal imaging

## Abstract

Emphysematous cholecystitis is a potentially life-threatening variant of acute cholecystitis, characterized by the presence of gas in the gallbladder wall/lumen due to the proliferation of gas-producing bacteria. Symptoms include upper right quadrant pain, fever, nausea, and vomiting. Laboratory tests may show nonspecific indications of systemic infection, and radiological assessment, especially CT scanning, is crucial for diagnosis. This case underscores the significance of early diagnosis and intervention in managing emphysematous cholecystitis to prevent serious complications and reduce the higher mortality rate compared to acute cholecystitis.

## Introduction

Emphysematous cholecystitis [EC] is a severe infectious condition of the gallbladder, occurring in approximately 1% of cases of acute cholecystitis [1]. First documented in 1896, EC has since been associated with gallbladder ischemia and invasion by gas-producing bacteria, with Clostridium perfringens and Escherichia coli identified as frequent causative agents [1], [2], [3]. Ischemia is believed to arise from compromised vascular supply to the gallbladder, often secondary to conditions such as atherosclerosis, arterial embolism, vasculitis, or systemic hypotension/hypoperfusion [3,4]. The resulting gallbladder necrosis creates an environment conducive to bacterial proliferation, leading to the characteristic gas accumulation inside the gallbladder [1,3].

## Case report

**Fig. 1:**
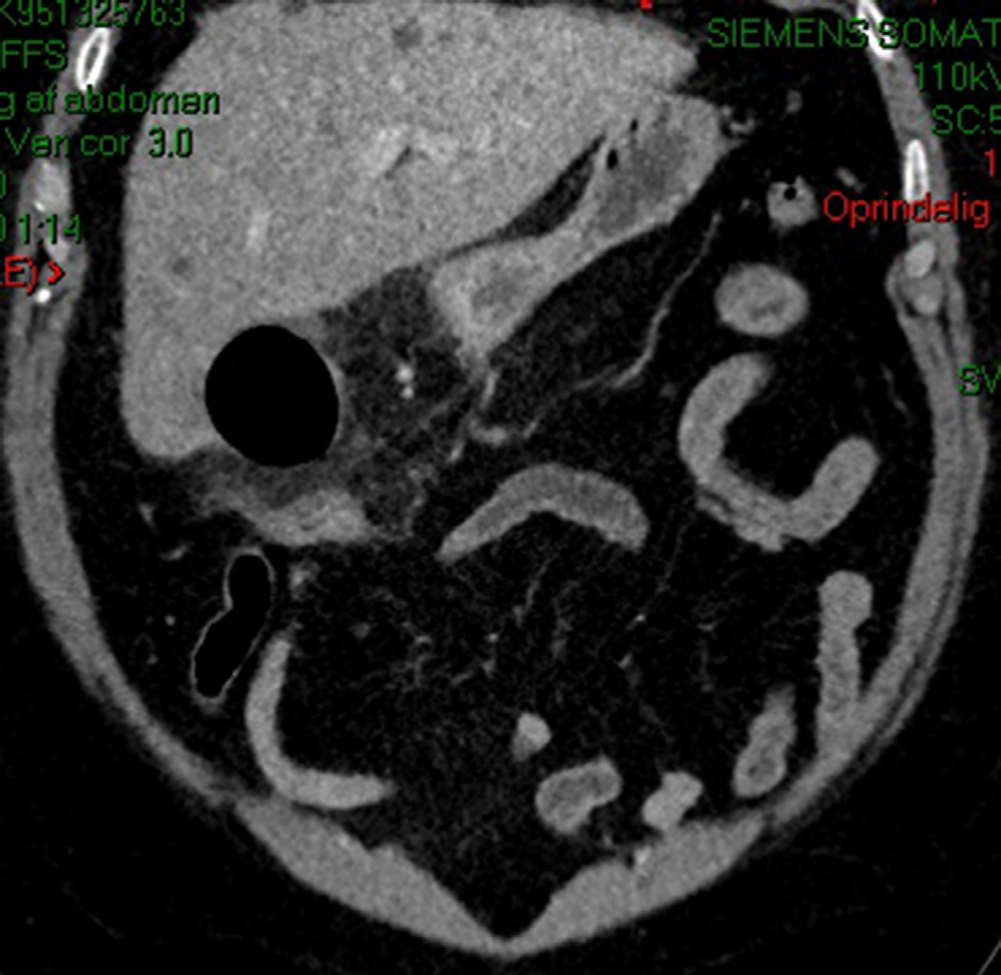
A contrast-enhanced CT scan coronal view showing acute emphysematous cholecystitis. Abnormally gas-filled gallbladder with inflammatory stranding of surrounding fat planes.

**Fig. 2:**
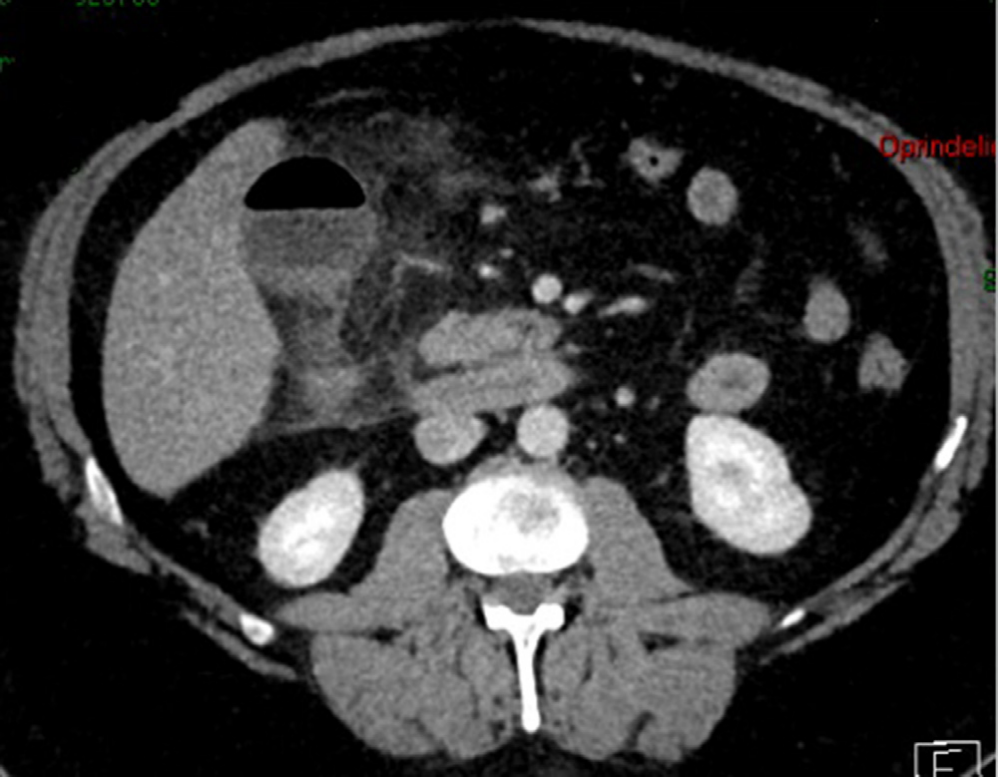
A contrast-enhanced CT scan axial view showing acute emphysematous cholecystitis. Air-fluid level in the lumen of gallbladder with wall thickening, increased enhancement in the gallbladder wall, inflammatory stranding of surrounding fat planes.

**Fig. 3:**
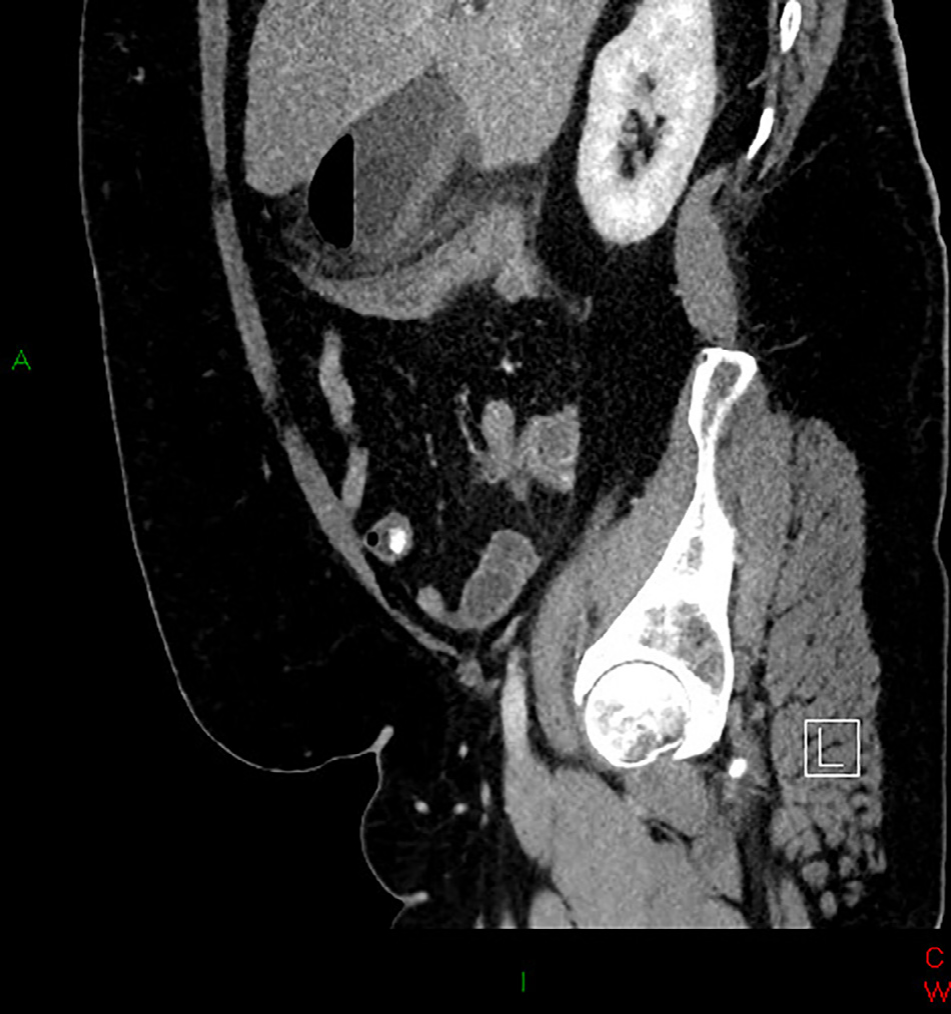
A contrast-enhanced CT scan sagittal plane showing acute emphysematous cholecystitis. Air-fluid level in the lumen of gallbladder with wall thickening, increased enhancement in the gallbladder wall, inflammatory stranding of surrounding fat planes.

A 57-year-old male presented at the emergency department with a 4-day history of persistent pain in the upper abdomen, initially attributed to reflux. There was an initial misdiagnosis due to the absence of troponin dynamics and normal biochemical markers during the first referral of the patient to the emergency department. Subsequent worsening of pain, particularly under the right upper quadrant, along with chest pain and shortness of breath, prompted a re-evaluation during the second referral to the emergency department. Notably, there was a single episode of yellow vomiting and normal bowel movements. On objective examination, direct tenderness under the right curvature in moderate to severe degree and percussion tenderness were observed. Laboratory findings showed leukocytosis (21.4 × 10^9^/L), neutrophilia (18.5 × 10^9^/L), elevated CRP (410 mg/L), and high plasma creatinine (111 micromoles/L), while liver parameters remained normal. The patient was also febrile (39.7°C) during this episode. The CT scan of the abdomen with IV contrast at the re-evaluation of the patient depicted gallbladder wall thickening, inflammatory reaction as fat stranding, along with abnormally gas filled gallbladder (Fig. 1) and an air-fluid level in the lumen (Figs. 2 and 3) consistent with acute emphysematous cholecystitis. The patient underwent laparoscopic cholecystectomy revealing a perforated and necrotic gallbladder which confirmed the diagnosis of Gangrenous Cholecystitis stemming from ischemia due to the gradual decline in vascular supply [4].

## Discussions

EC represents a rare and potentially life-threatening variant of acute cholecystitis, characterized by the presence of gas in the gallbladder wall / lumen [2]. Based on existing data, EC is more commonly observed in individuals in their sixth and seventh decades of life, with a higher prevalence among diabetic patients, constituting over half of the cases. In contrast to typical acute cholecystitis, EC exhibits a higher incidence in men, with a male-to-female ratio of 7:3 [1]. Patients affected by EC may experience symptoms such as pain in the upper right quadrant, accompanied by fever, nausea, and vomiting [3]. However, in some patients, there might be a lack of symptoms [1]. Laboratory tests are often nonspecific and may reveal only indirect indications of acute systemic infection, such as a leukocytosis [1]. Radiological assessment is crucial for diagnosis and serves as the primary method for achieving a proper preoperative diagnosis. The presence of gas in the gallbladder, as demonstrated through plain abdominal X-ray, ultrasound scan, or CT scan, is indicative of emphysematous cholecystitis [1]. Plain X-rays are not typically employed for diagnosing gallbladder disease. However, they may reveal abnormal gas formation within the gallbladder tissue, air-fluid levels, and curved lines of gas in the organ's wall [1].

In the majority of cases, the initial imaging study utilized when there is a clinical suspicion of acute cholecystitis is an ultrasound scan. Abnormal sonographic findings may be evident even before gas becomes visible in plain X-ray films. These findings encompass reverberation, or the artifact known as a “comet's tail,” resulting from an abrupt change in acoustic impedance at the soft tissue-gas interface [1]. Abdominal CT scanning is the most sensitive imaging modality for diagnosing emphysematous cholecystitis [2]. The radiological characteristics of EC can be categorized into three stages: the presence of gas inside the gallbladder (Stage 1), gas in the gallbladder wall (Stage 2), and gas in the adjacent tissues/ the pericholecystic fluid (Stage 3) [1,3]. timely diagnosis is crucial to minimize the associated mortality rates of EC [3]. Treatment of EC involves options such as laparoscopic or open cholecystectomy, percutaneous cholecystostomy, conservative therapies, or hyperbaric oxygen therapy [3].

## Conclusion

This case highlights the critical importance of early diagnosis and intervention in managing EC to reduce the risk of serious complications, such as perforation and sepsis. This is particularly crucial, given the higher mortality rate (15%-20%) compared to acute cholecystitis (4%) [3].

## Patient consent

Patient consent Informed consent for participation in the study and publication was obtained from the patient.
